# Tree seedling richness, but not neighborhood composition, influences insect herbivory in a temperate deciduous forest community

**DOI:** 10.1002/ece3.2336

**Published:** 2016-08-12

**Authors:** Stephen J. Murphy, Kaiyang Xu, Liza S. Comita

**Affiliations:** ^1^ Department of Evolution, Ecology and Organismal BiologyThe Ohio State University318 W. 12th AvenueColumbusOhio43210‐1293; ^2^ School of Forestry and Environmental StudiesYale University195 Prospect StreetNew HavenConnecticut06511; ^3^ Smithsonian Tropical Research InstituteBox 0843‐03092BalboaAncónPanama

**Keywords:** Density dependence, diversity, herd immunity, Janzen–Connell, mixed model, resource concentration, seedling dynamics

## Abstract

Insect herbivores can serve as important regulators of plant dynamics, but their impacts in temperate forest understories have received minimal attention at local scales. Here, we test several related hypotheses about the influence of plant neighborhood composition on insect leaf damage in southwestern Pennsylvania, USA. Using data on seedlings and adult trees sampled at 36 sites over an approximately 900 ha area, we tested for the effects of total plant density, rarefied species richness (i.e., resource concentration and dietary‐mixing hypotheses), conspecific density (i.e., Janzen–Connell hypothesis), and heterospecific density (i.e., herd‐immunity hypothesis), on the proportion of leaf tissue removed from 290 seedlings of 20 species. We also tested for the effects of generic‐ and familial‐level neighborhoods. Our results showed that the proportion of leaf tissue removed ranged from zero to just under 50% across individuals, but was generally quite low (<2%). Using linear mixed models, we found a significant negative relationship between insect damage and rarefied species richness, but no relationship with neighborhood density or composition. In addition, leaf damage had no significant effect on subsequent seedling growth or survival, likely due to the low levels of damage experienced by most individuals. Our results provide some support for the resource concentration hypothesis, but suggest a limited role for insect herbivores in driving local‐scale seedling dynamics in temperate forest understories.

## Introduction

The relationship between local plant diversity and herbivore damage has a long history in both agricultural and ecological research (Pimentel 1961). The idea that plant monocultures support high herbivore population densities dates back to at least the early 20th century (Marchal 1908), and the recognition of this relationship in natural systems was made soon after (Graham 1915; Pimentel 1961). This link between plant diversity and herbivory was first formalized by Root (1973) when he introduced the resource concentration hypothesis, positing that higher plant species richness leads to lower per‐species densities, thus limiting the total amount of resources available for specialist insect herbivores. As a result, high‐diversity stands are predicted to have lower insect herbivore loads than low diversity stands.

While the resource concentration hypothesis has been important in both ecological research and agricultural management, a recent review of the literature revealed only moderate overall support for the expected negative relationship between plant species richness and insect herbivore load/damage (Dinnage 2013). Specifically, Dinnage (2013) reported an equal number of studies showing a nonsignificant, or even positive, relationship between diversity and insect damage as did studies showing the expected negative relationship. Such positive relationships between diversity and damage are often thought to be a result of dietary mixing, where generalist herbivores benefit from feeding on a wide variety of host plant resources (Bernays et al. 1994). Thus, the diversity–herbivory relationship is thought to be context‐dependent, being co‐driven by factors such as the composition of the plant community (Vehviläinen et al. 2006), the composition of the insect community (especially the relative abundance of specialist versus generalist herbivores; Schuldt et al. 2010), and through variation in environmental resource gradients such as light and soil nutrients (Dinnage 2013).

A related, yet more mechanistic, hypothesis was proposed by Janzen (1970) and Connell (1971), who suggested that herbivore pressure should be positively correlated with conspecific neighbor densities (and/or negatively correlated with distance to conspecific neighbor), and not necessarily with species richness per se. As before, this is expected to result from specialist insect herbivores being attracted to high‐density patches of preferred forage. Conversely, the herd immunity hypothesis focuses on variation in heterospecific neighbor densities, predicting that large heterospecific patches confer protection from natural enemies by making it more difficult for specialists to locate host plants (Wills et al. 1997). Both the Janzen–Connell and herd immunity hypotheses are useful because they directly link herbivore pressure to plant demography and, ultimately, to species coexistence and diversity maintenance. Specifically, the variation in damage driven by plant neighborhood composition and density is expected to impact growth and survival (Hulme 1996). Therefore, individual plant fitness should be reduced in high conspecific stands and enhanced in high heterospecific stands.

Recently, neighborhood models have expanded the concept of the plant neighborhood proposed by the Janzen–Connell and herd immunity hypotheses to include not only con‐ and heterospecific densities, but also higher order taxonomic levels, such as congeneric and confamilial densities (e.g., Queenborough et al. 2009). Insect herbivores likely do not respond to species identity per se, but instead to individual plant traits such as leaf thickness, chemical defenses, and nutrient content. Thus, phylogenetically conserved traits that are associated with insect herbivory may result in broader host ranges (Ali and Agrawal 2012). In fact, few insect herbivores are known to be true monophages, with many being limited at the level of plant genera, rather than species (Basset 1992; Novotny et al. 2010). Thus, analyses that consider the density of generic or even familial neighbors may be more biologically relevant than analyses that only test for species‐level effects.

Despite widespread research on local‐scale insect herbivory in tropical forests, temperate grasslands, and agricultural systems, the impacts of insect herbivores on the local dynamics of temperate deciduous forests have received much less attention. Instead, most of the research on insect herbivory in temperate forests has either dealt with very large spatiotemporal scales (i.e., insect outbreaks) or has focused on invasive exotic insects. In comparison, little is known about local patterns of insect herbivore damage or the consequences of such damage for tree seedlings. This distinction may be driven by the idea that herbivory rates are lower in temperate regions than in the tropics (Coley and Aide 1991). However, recent work has indicated that the impacts of herbivores at localized scales may be just as important in temperate regions as in the tropics (Lambers et al. 2002), and actual evidence for a latitudinal gradient in herbivory remains conflicted (Andrew and Hughes 2005; Moles et al. 2011). Furthermore, the presence of herbivory on trees in this region is well known, including on small understory seedlings (Dudt and Shure 1994; Marquis and Whelan 1994; Barnes et al. 1998; Sobek et al. 2009), but the consequences of such damage remain largely unknown. Thus, more work is needed to better understand the role of insect herbivores in regulating local patterns and dynamics in forest understory communities.

In this study, we address two key questions related to standing insect herbivore damage on tree seedlings in a temperate forest in southwestern Pennsylvania. We ask (1) does the local density, diversity, and/or composition of neighboring plants influence the amount of herbivore damage a seedling experiences? We test several related hypotheses to address this question: (a) insect herbivore damage is correlated with total plant density, (b) insect herbivore damage is either negatively (i.e., resource concentration hypothesis) or positively (i.e., dietary mixing hypothesis) correlated with local species richness, (c) insect damage is positively correlated with local conspecific density (i.e., Janzen–Connell hypothesis), (d) insect herbivore damage is negatively correlated with local heterospecific density (i.e., herd immunity hypothesis), and (e) insect herbivore damage is negatively correlated with the local density of congeneric and confamilial neighbors. Secondly, we ask (2) does herbivore damage negatively impact plant fitness by reducing seedling growth and survival? While several studies have linked herbivore damage rates to plant neighborhood composition, few have studied the long‐term demographic effects of such damage. This is an important oversight, as it is through demographic processes such as growth and survival that herbivores should be capable of regulating local community dynamics.

## Materials and Methods

### Study area and species

The study was conducted at Powdermill Nature Reserve (PNR), an approximately 900‐ha area located in southwestern Pennsylvania (Westmoreland County) at the eastern edge of the Allegheny Plateau (40°09′N, 79°16′W). The area has a highly seasonal continental climate with annual precipitation around 1100 mm, and average monthly temperatures ranging from −7°C in January to 29°C in July and August. The vegetation of PNR is best described as mixed mesophytic, with maple (*Acer* spp.) and tulip poplar (*Liriodendron tulipifera*) dominating both the canopy and understory. However, other more xeric species such as oak (*Quercus* spp.) and hickory (*Carya* spp.) are also commonly found. Most of the forest at PNR is late successional (>80 years old), but some areas were cleared for mining operations in the 1940s or were used for agriculture up until the 1950s (Utech 1999). The current study focuses only on these late‐successional stands.

### Study and sampling design

**Figure 1:**
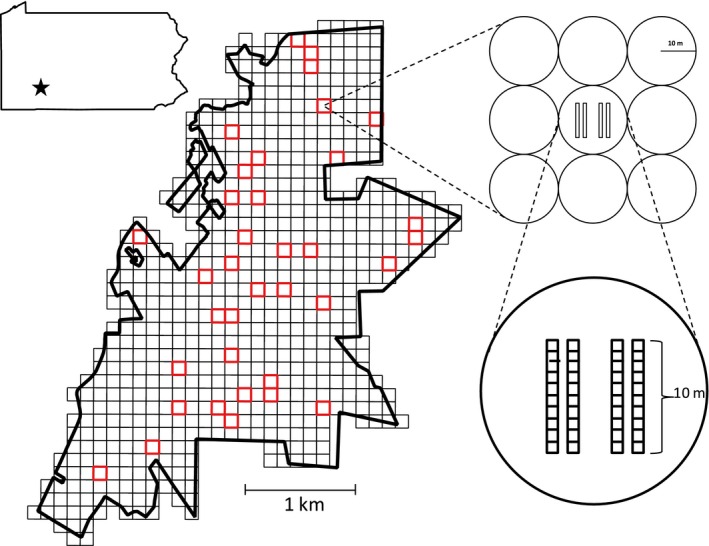
The location (black star) and boundary of Powdermill Nature Reserve (PNR) in Rector, Pennsylvania, showing the sampling design of the 2008 adult tree survey. A total of 647 120 × 120 m grid cells were used to cover the entire area, within which all trees ≥8 cm DBH were sampled within nine adjacent circular plots, each with a radius of 10 m. Within 45 randomly selected 120 × 120 m grid cells, four 10 × 1 m belt transects were established in 2012 to sample seedlings ≥7 cm tall. From these 45 plots, 36 contained seedlings ≥20 cm where photographs were taken to quantify herbivore damage (red outline).

In 2008, a large‐scale vegetation survey was conducted at PNR to document the distribution and abundance of trees and shrubs across the entire reserve. A total of 647 quadrats were sampled on a grid and permanently marked using steel rebar (Fig. 1; Murphy et al. 2015). At these locations, nine adjacent circular plots of 10 m radius were sampled. All adult trees ≥8 cm diameter at breast height (DBH) were identified and measured, which we used to calculate adult neighborhood metrics. In 2013, we randomly selected a subset of 45 of the larger quadrats to sample the understory seedling layer in more detail. Only quadrats that were within mature forest (as estimated using historical aerial photographs from 1939) were used in our random sample (see Murphy et al. 2015 for details). Within these quadrats, four 10 × 1 m belt transects were established to sample woody seedlings (Fig. 1). Each belt transect was divided into 10 individual 1 × 1 m subquadrats, and all seedlings ≥7 cm tall were tagged, identified, and measured for height. Sampling occurred from June to August 2013 and from July to August 2014. Data from the 2013 seedling census were used to calculate seedling neighborhood metrics as described below in more details.

### Measuring herbivore damage

Data on standing insect herbivore damage were collected on a subset of the seedlings marked in the 2013 census. Only seedlings ≥20 cm tall from deciduous species were used to estimate herbivore damage, and a maximum of five individuals, regardless of species, was randomly sampled per 10 × 1 m transect. Nine of the 45 large quadrats that we sampled did not contain any seedlings >20 cm, resulting in a total quadrat sample of 36 (Fig. 1). Our sampling design resulted in 290 individual seedlings from 20 species. These species represent a good representation of the overall PNR tree community, as well as the general composition of western Pennsylvanian forests (Appendix S1; Utech 1999; Murphy et al. 2015). It is important to note that our goal was to assess the drivers and impacts of herbivory at the community level. Thus, our sampling scheme for herbivore damage measurements resulted in variable sample sizes among the 20 species, reflecting their relative abundances in the overall understory community at PNR and precluding any analyses at the individual species level (Appendix S1).

We assessed herbivore damage by photographing leaves of the focal seedlings under overcast sky conditions in the field. An opaque parasol was used to further reduce reflectance from the sun. A white foam board was placed behind the leaves, and a thin piece of antireflective plexiglass was placed overtop to ensure a flat surface for photographing. Enough photographs were taken to sample all leaves of an individual, or up to five randomly sampled pictures, whichever came first (less than 5% of all individuals sampled required more than five pictures to sample all leaves). All pictures were taken using an Olympus Evolt‐220 digital camera during the last week of July in 2013. Photographs were then imported into the imaging software Image‐J (Rasband 2015). Images were split into red, green, and blue color channels to better isolate vegetation from nonvegetation areas. Contrast between leaf and nonleaf areas of the photographs was further enhanced via image segmentation using the threshold function. Images were then converted to binary (i.e., black and white pixels only), and any additional nonleaf portions of the image, including woody stems, were removed by hand using the eraser function. Total leaf area was then calculated using these modified images. If the margin of a leaf was compromised due to herbivore damage, the paint tool was used to estimate the true margin. Finally, the total area of removed leaf tissue was calculated, and the total proportion of leaf damage was calculated for each seedling as area of removed leaf tissue/total leaf area.

Our method of quantifying insect damage was able to account for a wide variety of damage types, including from leaf mining and skeletonizing insects. However, damage types that resulted in a significant amount of remaining photosynthetic tissue (e.g., aphid suckering damage) may be underestimated using this approach.

### Data and statistical analysis

**Figure 2:**
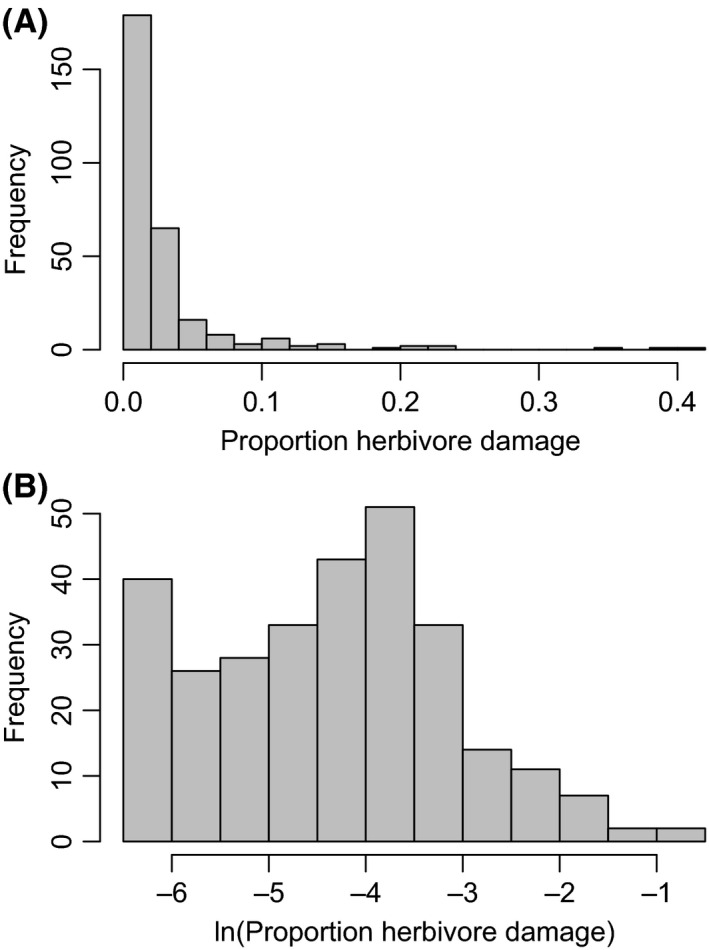
Histograms showing the proportion of leaf damage (A) and ln‐transformed proportion of leaf damage (B) for 290 tree seedlings sampled. Proportion of leaf damage was estimated from digital photographs collected in the field.

The proportion of leaf damage across individuals was highly right skewed and required a ln‐transformation to meet statistical assumptions (Fig. 2). Furthermore, because the data contained many zeros (i.e., no insect damage observed), we rescaled the data using the equation:

(1) $$x^{\prime }=\frac{x(N-1)+0.5}{N}$$

where *x* is the proportion of leaf damage and *N* is the total number of samples (Smithson and Verkuilen 2006). In all subsequent analyses, the ln‐transformation of *x*’ was used. We then fit linear mixed‐effects models to assess the relationship between insect leaf damage and the density, richness, and composition of local seedling neighbors found in the same 1 × 1 m subquadrat as the focal seedlings. Our first model tested the effects of total plant neighborhood density, independent of the species composition of the neighborhood (i.e., density model). Next, we tested the relationship between species richness and damage (i.e., resource concentration and dietary mixing model). Under the resource concentration hypothesis, we would expect a negative relationship between rarefied richness and damage, while under dietary mixing, we would expect a positive relationship. Actual species richness varied from 1 to 8 at the 1 × 1 m scale, and from 3 to 24 at the 20 × 20 m scale. However, because we did not want the effects of total plant density to confound the effects of species richness (Gotelli and Colwell 2001), we used rarefied species richness instead of raw counts. This was carried out by taking the average value of repeated random samples of 2 individual seedlings from each quadrat. Thus, rarefied species richness was bounded between 1 and 2.

To test the Janzen–Connell and herd immunity hypotheses in our study system, we ran a model that included conspecific and heterospecific seedling neighbor densities. Under the Janzen–Connell hypothesis, we would expect a positive correlation between damage and conspecific density, and under the herd immunity hypothesis, we would expect to see a negative correlation between damage and heterospecific density. Finally, we ran a model in which we replaced con‐ and heterospecific seedling densities with con‐ and heterogeneric and con‐ and heterofamilial neighborhood densities to detect neighborhood effects of insects specialized at the genus or family level, respectively.

To assess whether leaf damage from insect herbivores was dependent on the scale of the plant neighborhood used, we also ran the models described above using plant neighborhood metrics computed using information on all seedlings sampled within all four 10 × 1 m belt transects in each quadrat. Finally, we tested whether the adult tree neighborhood community had an influence on the amount of insect damage experienced by tree seedlings. All trees previously sampled within the central circular plot (i.e., 314 m^2^) were used to calculate adult neighborhood metrics using tree basal area. We also reran this analysis using all nine survey plots (i.e., 2826 m^2^).

Lastly, the census data collected in 2013 and 2014 were used to test for an effect of herbivore damage on individual seedling growth and survival. Growth was modeled as ln‐transformed relative growth rate [(ln(Height_2014_) − ln(Height_2013_)] using a linear mixed model. Survival (i.e., dead or alive after 1 year) was modeled using a generalized linear mixed model (GLMM) with a Bernoulli error distribution. Both the growth and survival models included ln‐transformed proportional leaf damage as the predictor variable.

In all of the regression models presented in this study (Tables 1, 2, and S2), we controlled for variation in seedling size by including ln‐transformed initial height as a covariate. We also included the 20 × 20 m block identity as a random effect to account for spatial autocorrelation. Likewise, species identity was treated as a random effect to control for potential variation among species in herbivore damage and growth and survival rates. Model comparison was conducted by reporting both the Akaike information criterion (AIC) and Bayesian information criterion (BIC). All analyses were carried out in the R statistical programming environment (R Core Team 2015). Linear mixed models were fit using ordinary least squares in the nlme package (Pinheiro et al. 2015), and GLMMs were fit using the lme4 package (Bates et al. 2014). Rarefied richness was calculated in the vegan package using the function rarefy (Oksanen et al. 2015).

**Table 1:** Linear mixed‐effects models relating neighbor density, composition, and rarefied species richness with proportion of leaf area lost due to herbivore damage. The first model set (1–5) uses plant neighborhood metrics calculated at the scale of 1 × 1 m^2^ seedling plots, while the second set (6–10) uses plant neighborhood metrics calculated using seedling data from all four 1 × 10 m^2^ belt transects within each block. The final model set (11–15) uses basal area of adult neighbors ≥8 cm, rather than density of seedling neighbors, to calculate neighbor metrics. All models included ln‐transformed seedling height as a covariate, as well as random block and species effects terms. Values in bold indicate significant terms, as well as best fit models based on AIC and BIC

	Model formula	Estimate	SE	*P*‐value	AIC	BIC
(A) 1‐m^2^ neighborhood	1) ln(Damage) ~ ln(Height) + Density					
	ln(Height)	0.512	0.970	0.077	980.9	1002.9
	Density	0.000	0.011	0.774		
	2) ln(Damage) ~ ln(Height) + Rarefied Richness					
	ln(Height)	0.463	0.281	0.102	**964.0**	**9856.0**
	Rarefied Richness	−0.796	0.238	**0.001**		
	3) ln(Damage) ~ ln(Height) + Conspecifics + Heterospecifics					
	ln(Height)	0.523	0.291	0.075	988.2	1013.8
	Conspecifics	0.009	0.026	0.722		
	Heterospecifics	0.002	0.013	0.883		
	4) ln(Damage) ~ ln(Height) + Congenerics + Heterogenerics					
	ln(Height)	0.520	0.292	0.077	988.3	1013.8
	Congenerics	0.007	0.025	0.772		
	Heterogenerics	0.002	0.013	0.860		
	5) ln(Damage) ~ ln(Height) + Confamilials + Heterofamilials					
	ln(Height)	0.522	0.292	0.077	988.2	1013.8
	Confamilials	0.008	0.025	0.744		
	Heterofamilials	0.002	0.013	0.882		
(B) 40‐m^2^ neighborhood	6) ln(Damage) ~ ln(Height) + Density					
	ln(Height)	0.493	0.287	0.088	987.1	1009.1
	Density	−0.000	0.000	0.456		
	7) ln(Damage) ~ ln(Height) + Rarefied Richness					
	ln(Height)	0.527	0.288	0.069	971.4	993.3
	Rarefied Richness	−0.870	0.707	0.222		
	8) ln(Damage) ~ ln(Height) + Conspecifics + Heterospecifics					
	ln(Height)	0.491	0.291	0.094	1000.5	1026.1
	Conspecifics	−0.001	0.001	0.745		
	Heterospecifics	−0.001	0.001	0.522		
	9) ln(Damage) ~ ln(Height) + Congenerics + Heterogenerics					
	Height	0.482	0.291	0.100	1000.5	1026.1
	Congenerics	−0.001	0.001	0.600		
	Heterogenerics	−0.001	0.001	0.582		
	10) ln(Damage) ~ ln(Height) + Confamilials + Heterofamilials					
	ln(Height)	0.490	0.291	0.094	1000.5	1026.1
	Confamilials	−0.001	0.001	0.720		
	Heterofamilials	−0.001	0.001	0.543		
(C) Adult tree neighborhood	11) ln(Damage) ~ ln(Height) + Basal Area					
	ln(Height)	0.506	0.287	0.080	980.4	1002.4
	Basal Area	−0.003	0.015	0.833		
	12) ln(Damage) ~ ln(Height) + Rarefied Richness					
	ln(Height)	0.465	0.289	0.109	974.3	996.2
	Rarefied Richness	0.198	0.164	0.231		
	13) ln(Damage) ~ ln(Height) + Conspecifics + Heterospecifics					
	Height	0.497	0.289	0.087	974.6	1000.2
	Conspecifics	−0.105	0.753	0.889		
	Heterospecifics	0.238	0.181	0.192		
	14) ln(Damage) ~ ln(Height) + Congenerics + Heterogenerics					
	ln(Height)	0.483	0.289	0.097	974.9	1000.5
	Congenerics	−0.171	0.514	0.739	
	Heterogenerics	0.265	0.182	0.149	
	15) ln(Damage) ~ ln(Height) + Confamilials + Heterofamilials					
	ln(Height)	0.505	0.286	0.080	974.4	1000.0
	Confamilials	−0.238	0.378	0.530		
	Heterofamilials	0.318	0.186	0.090		

**Table 2:** Linear mixed‐effects models relating relative growth and survival to the proportion of leaf damage observed. Both models include plot and species random effects to control for spatial autocorrelation and potential differences among species

Model formula	Estimate	Standard error	*P*‐value
1) Relative growth rate ~ ln(Height) + ln(Damage)			
ln(Height)	0.018	0.086	0.665
ln(Damage)	0.006	0.017	0.7421
2) Survival ~ ln(Height) + ln(Damage)			
ln(Height)	0.262	0.593	0.658
ln(Damage)	−0.053	0.123	0.667

## Results

Proportion of standing leaf damage from insect herbivory ranged from 0.0 to 0.46 among the 290 individuals sampled (Fig. 2). These data were highly right skewed, with most individuals having low levels of herbivore damage (median community‐wide proportional leaf damage was 0.012, vs. a mean of 0.027). Forty‐six individuals had no observable insect damage. There were also noticeable patterns in the within and among species damage rates observed across the 20 species analyzed (Fig. S1). *Tilia americana* had the highest levels of median and maximal damage. In addition to *T. americana*, two *Nyssa sylvatica* seedlings also experience very high levels of herbivory (i.e., >35%). Other species tended to have consistently low levels of leaf damage.

**Figure 3:**
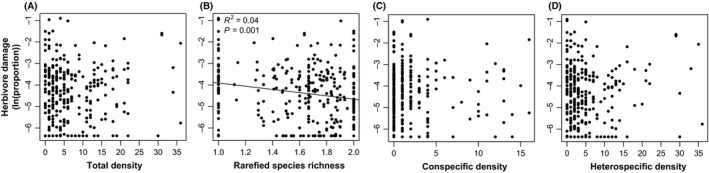
Relationships between herbivore damage (ln(proportion)) and four neighborhood variables: (A) total seedling density, (B) rarefied species richness, (C) conspecific seedling density, and (D) heterospecific seedling density. The black line in panel (B) indicates the significant best‐fit regression line, and *R* ^2^ and *P* values are shown in the top left.

Of the fifteen models testing for a relationship between herbivore damage and neighborhood metrics, the best‐fit model was the one with rarefied species richness at the 1‐m^2^ scale (Table 1). In fact, rarefied species richness was the only neighborhood variable that was significantly related to herbivore damage, with increasing species richness of seedling neighbors resulting in lower herbivore damage (Fig. 3, Table 1). However, the amount of variation in herbivore damage explained by rarefied species richness alone was relatively low (*R* ^2^ = 0.04, Fig. 3). Furthermore, when using all seedlings sampled within a quadrat (i.e., all four belt transects used), rarefied species richness was no longer significant (Table 1B), indicating that the effect occurred over a very small spatial scale (i.e., 1‐m^2^).

Variation among individuals in herbivore damage was unrelated to the density of neighboring conspecific and heterospecific seedlings at either spatial scale (Table 1A,B). Likewise, there were no significant effects of neighborhood densities based on adult basal area (Table 1, Table S2), nor when replacing species‐level neighborhood densities with genus‐ and family‐level metrics. Lastly, we found no effect of plant herbivore damage on seedling relative growth rates or probability of survival over the 1‐year study interval (Table 2).

## Discussion

### Community‐wide variation in leaf damage

We found insect herbivore damage to be generally low within the understory community at Powdermill Nature Reserve (PNR). While some individuals experienced close to 50% leaf damage, the majority had less than 2% of leaf tissue removed (Fig. 2). These numbers are generally in line with other studies reporting leaf tissue removal rates in temperate forest understories (Dudt and Shure 1994; Marquis and Whelan 1994; Barnes et al. 1998; Sobek et al. 2009). The reason for these relatively low rates of damage is often thought to be due to lower herbivore densities in temperate regions compared to tropical systems, coupled with lower degrees of specialization Coley and Aide 1991). Thus, it is overall not surprising that we recorded such low damage rates here. At the same time, our snapshot measure of damage represents the net impacts of insect herbivores over the lifespan of individual leaves. Thus, we were unable to determine temporal variation in damage rates, nor could we account for the possibility of whole‐leaf consumption. Therefore, our estimates of insect damage are likely an underestimate of the true pressures that understory seedlings suffer in these forests. Further studies tracking individual leaves through time would be helpful to further elucidate these patterns.

### Neighborhood diversity, composition, and insect herbivory

Our results provide some support for the resource concentration hypothesis. We found a significant, yet weak, negative relationship between rarefied species richness and standing herbivore damage, which suggests that increasing plant species richness decreases insect pressure on plants. Our results, however, indicate that the relationship between richness and herbivory was not driven by the abundance of conspecific or heterospecific neighbors. This pattern remained true when using generic and familial neighborhoods as well. Both the Janzen–Connell and herd immunity hypotheses should result in significant correlations between leaf damage and plant neighborhood densities if insect herbivores are an important mechanistic driver of these interactions. Thus, we found no support for either of these hypotheses. At the same time, Janzen–Connell interaction can be driven by distances from adult neighbors in addition to the densities of the local seedling neighborhood. Because our sampling design did not allow for the calculation of distances between adults and seedlings, we were unable to address this important aspect of the Janzen–Connell hypothesis. However, because conspecific seedling density and distance from adult neighbor tend to be highly correlated, we do not expect there to be a significant difference from what was observed here if distance effects were analyzed.

It is also worth mentioning that the historical view that richness is simply a resultant property of individual species interactions has been challenged in recent decades (Tilman and Downing 1994). For herbivores, it is possible that local community richness itself influences the behavior of insect herbivores, instead of through an indirect effect on local plant species densities. For example, it may be more difficult to locate host tissue when many different nonhosts, regardless of density, surround the plant. Alternatively, there may be an additional unmeasured factor (e.g., nutrient availability) that is driving spatial variation in both plant species richness and herbivore damage rates at our study site. Regardless, our results do point to the necessity of quantifying neighborhood composition when conducting studies on diversity–herbivory relationships, instead of looking at local diversity metrics alone. Experimental manipulations that can tease apart density‐ versus frequency‐dependent neighborhood interactions would be productive for exploring these dynamics further (Hambäck et al. 2014).

We were also unable to assess the effects of more complex metrics of plant neighborhood similarity, such as phylogenetic distance among individuals, which may mask the role of neighbor relatedness in our study. Dinnage (2013), testing the resource concentration hypothesis in an old field community, found mean phylogenetic distance of neighboring plants to be negatively correlated with herbivore damage. Interestingly, he found the exact opposite relationship when using species richness, suggesting that insect herbivores may respond to plant neighborhood composition in complex ways. Phylogenetic neighborhood has also been shown to impact plant growth and survival, primarily in tropical systems (Paine et al. 2011). At the same time, others have suggested no relationship (Uriarte et al. 2010), or even a negative relationship (Zhu et al. 2015), between phylogenetic distance of neighbors and plant performance. Likewise, functional trait diversity has similarly been used to test the Janzen–Connell hypothesis and has resulted in mixed conclusions overall (Uriarte et al. 2010; Paine et al. 2011; Kunstler et al. 2012). There may also be important differences in the strength of plant–herbivore interactions among individual species that we were unable to adequately quantify in our models due to low sample sizes. Future studies that explicitly focus on such interspecific differences would be useful for assessing community‐wide variability in plant–herbivore relationships.

Finally, the composition of the insect community itself is an important factor that we were unable to account for in the current analysis. Support for both the resource concentration hypothesis and Janzen–Connell hypothesis requires insect host specificity at some taxonomic level, whether it be at the species, genus, or family level. Alternatively, if the insect community at PNR is comprised predominately of generalist herbivores, then we would not expect to see a negative correlation between diversity and damage. Instead, generalist herbivores may be unaffected by local diversity or composition, or they may actually benefit from feeding within high‐diversity stands by providing a broader suite of essential resources (i.e., dietary mixing hypothesis). Therefore, the strength and direction of the diversity–herbivory relationship is likely influenced strongly by the degree of specialization of the local insect community. As it has been suggested that insects are less specialized in temperate forests than in the tropics, exploring these interactions across latitudinal or environmental gradients would be productive. Finally, understanding variation in the types of insect herbivores causing leaf tissue removal would also be helpful for unraveling these interactions (Bachelot and Kobe 2013).

### Insect herbivore effects on plant fitness

We found no correlation between standing insect herbivore damage and plant performance, whether measured by relative growth rate or by survival over 1 year. This was likely due to the low rates of leaf damage that we observed. However, other studies have indicated that insect herbivory can have large consequences for plant fitness, even in nonoutbreak years, by directly impacting plant performance through removal of leaf tissue (Hulme 1996). In a tropical community, Eichorn et al. (2010) showed that increasing rates of damage resulted in an increased risk of mortality, although growth rates were unaffected. Overall, however, surprisingly few studies to our knowledge have linked insect leaf damage with specific demographic processes such as growth, survival, or reproduction.

In our study, damage from insects may simply be too low to reduce plant performance significantly. Several studies have suggested that in the eastern deciduous forests, insect damage only rarely results in individual plant mortality (Baker 1972), except during rare and severe outbreak events (Kurz et al. 2008). In these systems, it is likely that other factors such as competition for light and soil resources play a stronger role in seedling demography than does leaf damage from insect herbivores. Incorporating such environmental gradients into our growth and mortality models could potentially reveal subtle impacts of herbivores. Finally, our study focused on drivers and impacts of insect herbivore damage at the whole community level. However, individual plant species undoubtedly vary in the amount and impact of herbivore damage experienced, with such variation likely driven by differences in life history strategy (e.g., shade tolerance). Thus, insect herbivory may play an important role for some species in the community, despites the overall weak effects we observed at the community level.

## Conclusions

In line with the results from Dinnage (2013), our study supports the notion that the relationship between local stand diversity and herbivore pressure is complex and may not be generalizable across communities or ecosystems. Our results do provide support for a significant, but very weak, relationship between local plant species richness and insect herbivore damage, consistent with the resource concentration hypothesis. However, overall our study suggests a limited role of insect herbivores in structuring the diversity and demography of temperate eastern deciduous forest seedling communities. Nonetheless, recent studies in the region have found a link between plant neighborhood composition and population dynamics, likely due to natural enemy attack (Johnson et al. 2012). Our findings indicate that future research should focus on mechanisms other than insect herbivores (e.g., fungal pathogens) as the drivers of neighborhood impact on plant performance.

## Conflict of Interest

None declared.

## Supporting information


**Table S1.** Rank abundance tables for adult trees, seedlings ≥7 cm in height, and the 290 seedlings sampled for herbivore damage.

**Table S2.** Linear mixed‐effects models relating adult neighbor basal area, composition and rarefied species richness with proportion of leaf area lost due to herbivore damage.

**Figure S1.** Boxplots showing the proportion of leaf tissue area removed or damaged from 290 sampled tree seedlings.

**Appendix S1.** Additional species‐level information for trees and seedlings.

**Appendix S2.** Model output from adult tree neighborhoods using all nine circular subplots.

## References

[ece32336-bib-0001] Ali, J. G. , and A. A. Agrawal . 2012 Specialist versus generalist insect herbivores and plant defense. Trends Plant Sci. 17:293–302. 22425020 10.1016/j.tplants.2012.02.006

[ece32336-bib-0600] Andrew, N. R. , and L. Hughes . 2005 Herbivore damage along a latitudinal gradient: relative impacts of different feeding guilds. Oikos. 108:176–182.

[ece32336-bib-0002] Bachelot, B. , and R. K. Kobe . 2013 Rare species advantage? Richness of damage types due to natural enemies increases with species abundance in a wet tropical forest. J. Ecol. 101:846–856.

[ece32336-bib-0003] Baker, W. 1972 Eastern forest insects. USDA Forest Service Misc. Publ. No. 1175. Washington, DC.

[ece32336-bib-0004] Barnes, B. , D. Zak , S. Denton , and S. Spurr . 1998 Forest ecology. 4th ed. John Wiley and Sons, New York, NY.

[ece32336-bib-0005] Basset, Y. 1992 Host specificity of arboreal and free‐living insect herbivores in rain forests. Biol. J. Linn. Soc. 47:115–133.

[ece32336-bib-0006] Bates, D. , M. Maechler , B. Bolker , and S. Walker . 2014 Fitting linear mixed‐effects models using lme4. J. Stat. Softw. 67:1–48.

[ece32336-bib-0007] Bernays, E. A. , K. L. Bright , N. Gonzalez , and J. Angel . 1994 Dietary mixing in a generalist herbivore: tests of two hypotheses. Ecology 75:1997–2006.

[ece32336-bib-0008] Coley, P. D. , T. M. Aide 1991 Comparison of herbivory and plant defences in temperate and tropical broad‐leaved forests Pp. 25–49 *in* Price P. W., Lewisohn T. M., Fernandes G. W., et al., eds. Plant‐animal interactions: evolutionary ecology in the tropical and temperate regions. Wiley & Sons, New York, NY.

[ece32336-bib-0009] Connell, J. H. 1971 On the role of natural enemies in preventing competitive exclusion in some marine animals and in rain forest trees Pp. 298–312 *in* den Boer P. and Gradwell G., eds. Dynamics of populations. Centre for Agricultural Publication and Documentation, Wagningen, the Netherlands.

[ece32336-bib-0010] Dinnage, R. 2013 Phylogenetic diversity of plants alters the effect of species richness on invertebrate herbivory. PeerJ 1:e93. 23825795 10.7717/peerj.93 PMC3698468

[ece32336-bib-0011] Dudt, J. F. , and D. J. Shure . 1994 The influence of light and nutrients on foliar phenolics and insect herbivory. Ecology 75:86–98.

[ece32336-bib-0012] Eichorn, M. P. , R. Nilus , S. G. Compton , S. E. Hartley , and D. F. R. P. Burslem . 2010 Herbivory of tropical rain forest tree seedlings correlates with future mortality. Ecology 91:1092–1101. 20462123 10.1890/09-0300.1

[ece32336-bib-0013] Gotelli, N. , and R. Colwell . 2001 Quantifying biodiversity: procedures and pitfalls in the measurement and comparison of species richness. Ecol. Lett. 4:379–391.

[ece32336-bib-0014] Graham, S. A. 1915 The biology and control of the white‐pine weevil (Pissodes strobi). Cornell University, Ithaca, NY.

[ece32336-bib-0015] Hambäck, P. A. , B. D. Inouye , B. D. Anderson , and N. Underwood . 2014 Effects of plant neighborhoods on plant–herbivore interactions: resource dilution and associational effects. Ecology 95:1370–1383. 25000768 10.1890/13-0793.1

[ece32336-bib-0016] Hulme, P. E. 1996 Herbivory, plant regeneration, and species coexistence. J. Ecol. 84:609–615.

[ece32336-bib-0017] Janzen, D. H. 1970 Herbivores and the number of tree species in tropical forests. Am. Nat. 104:501–528.

[ece32336-bib-0018] Johnson, D. J. , W. T. Beaulieu , J. D. Bever , and K. Clay . 2012 Conspecific negative density dependence and forest diversity. Science 336:904–907. 22605774 10.1126/science.1220269

[ece32336-bib-0019] Kunstler, G. , S. Lavergne , B. Courbaud , W. Thuiller , G. Vieilledent , N. E. Zimmermann , et al. 2012 Competitive interactions between forest trees are driven by species’ trait hierarchy, not phylogenetic or functional similarity: implications for forest community assembly. Ecol. Lett. 15:831–840. 22625657 10.1111/j.1461-0248.2012.01803.x PMC4003531

[ece32336-bib-0020] Kurz, W. A. , C. C. Dymond , G. Stinson , G. J. Rampley , E. T. Neilson , A. L. Carroll , et al. 2008 Mountain pine beetle and forest carbon feedback to climate change. Nature 452:987–990. 18432244 10.1038/nature06777

[ece32336-bib-0021] Lambers, J. H. R. , J. S. Clark , and B. Beckage . 2002 Density‐dependent mortality and the latitudinal gradient in species diversity. Nature 417:732–735. 12066182 10.1038/nature00809

[ece32336-bib-0022] Marchal, P. 1908 The utilization of auxiliary entomophagous insects in the struggle against insects injurious to agriculture. Pop. Sci. Mon. 72:352–419.

[ece32336-bib-0023] Marquis, R. , and C. Whelan . 1994 Insectivorous birds increase growth of white oak through consumption of leaf‐chewing insects. Ecology 75:2007–2214.

[ece32336-bib-0024] Moles, A. T. , S. P. Bonser , A. G. B. Poore , I. R. Wallis , and W. J. Foley . 2011 Assessing the evidence for latitudinal gradients in plant defence and herbivory. Funct. Ecol. 25:380–388.

[ece32336-bib-0025] Murphy, S. J. , L. D. Audino , J. Whitacre , J. L. Eck , J. W. Wenzel , S. A. Queenborough , et al. 2015 Species associations structured by environment and land‐use history promote beta‐diversity in a temperate forest. Ecology 96:705–715. 26236867 10.1890/14-0695.1

[ece32336-bib-0026] Novotny, V. , S. E. Miller , L. Baje , S. Balagawi , Y. Basset , L. Cizek , et al. 2010 Guild‐specific patterns of species richness and host specialization in plant‐herbivore food webs from a tropical forest. J. Anim. Ecol. 79:1193–1203. 20673235 10.1111/j.1365-2656.2010.01728.x

[ece32336-bib-0027] Oksanen, J. , F. Guillaume Blanchet , R. Kindt , P. Legendre , P. R. Minchin , R. B. O'Hara , et al. 2015 vegan: community ecology package. R package version 2.2‐1. http://CRAN.R-project.org/package=vegan

[ece32336-bib-0028] Paine, C. E. T. , N. Norden , J. Chave , P. M. Forget , C. Fortunel , K. G. Dexter , et al. 2011 Phylogenetic density dependence and environmental filtering predict seedling mortality in a tropical forest. Ecol. Lett. 15:34–41. 22004454 10.1111/j.1461-0248.2011.01705.x

[ece32336-bib-0029] Pimentel, D. 1961 Species diversity and insect population outbreaks. Ann. Entomol. Soc. Am. 54:76–86.

[ece32336-bib-0030] Pinheiro, J. , D. Bates , S. DebRoy , and D. Sarkar , and R Core Team . 2015 nlme: linear and nonlinear mixed effects models. R package version 3.1‐122, http://CRAN.R-project.org/package=nlme

[ece32336-bib-0031] Queenborough, S. A. , D. F. R. P. Burslem , N. C. Garwood , and R. Valencia . 2009 Taxonomic scale‐dependence of habitat niche partitioning and biotic neighbourhood on survival of tropical tree seedlings. Proc. R. Soc. B 276:4197–4205. 10.1098/rspb.2009.0921 PMC2821336 19740886

[ece32336-bib-0032] R Core Team . 2015 R: a language environment for statistical computing. R Foundation for Statistical Computing, Vienna, Austria URL http://www.R-project.org/

[ece32336-bib-0033] Rasband, W. S. 2015 ImageJ Software. U. S. National Institutes of Health, Bethesda, MD, http://imagej.nih.gov/ij/, 1997–2015

[ece32336-bib-0034] Root, R. B. 1973 Organization of a plant‐arthropod association in simple and diverse habitats: the fauna of collards (*Brassica oleracea*). Ecol. Monogr. 43:95–124.

[ece32336-bib-0035] Schuldt, A. , M. Baruffol , M. Böhnke , H. Bruelheide , W. Härdtle , A. C. Lang , et al. 2010 Tree diversity promotes insect herbivory in subtropical forests of south‐east China. J. Ecol. 98:917–926. 20852667 10.1111/j.1365-2745.2010.01659.x PMC2936109

[ece32336-bib-0036] Smithson, M. , and J. Verkuilen . 2006 A better lemon squeezer? Maximum‐likelihood regression with beta‐distributed dependent variables. Psychol. Methods 11:54–71. 16594767 10.1037/1082-989X.11.1.54

[ece32336-bib-0037] Sobek, S. , C. Scherber , I. Steffan‐Dewenter , and T. Tscharntke . 2009 Sapling herbivory, invertebrate herbivores and predators across a natural tree diversity gradient in Germany's largest connected deciduous forest. Oecologia 160:279–288. 19238448 10.1007/s00442-009-1304-2 PMC3085765

[ece32336-bib-0038] Tilman, D. , and J. Downing . 1994 Biodiversity and stability in grasslands. Nature 367:363–365.

[ece32336-bib-0039] Uriarte, M. , N. G. Swenson , R. L. Chazdon , L. S. Comita , W. J. Kress , D. Erickson , et al. 2010 Trait similarity, shared ancestry and the structure of neighbourhood interactions in a subtropical wet forest: implications for community assembly. Ecol. Lett. 13:1503–1514. 21054732 10.1111/j.1461-0248.2010.01541.x

[ece32336-bib-0040] Utech, F. H. 1999 Checklist of the vascular plants of Powdermill Nature Reserve, Westmoreland County, Pennsylvania. Carnegie Museum of Natural History, special publication No. 20, Pittsburgh, PA.

[ece32336-bib-0041] Vehviläinen, H. , J. Koricheva , K. Ruohomäki , T. Johansson , and S. Valkonen . 2006 Effects of tree stand species composition on insect herbivory of silver birch in boreal forests. Basic Appl. Ecol. 7:1–11.

[ece32336-bib-0042] Wills, C. , R. Condit , R. B. Foster , and S. P. Hubbell . 1997 Strong density‐ and diversity‐related effects help to maintain tree species diversity in a neotropical forest. Proc. Natl Acad. Sci. USA 94:1252–1257. 11038601 10.1073/pnas.94.4.1252 PMC19777

[ece32336-bib-0043] Zhu, Y. , L. S. Comita , and S. P. Hubbell . 2015 Conspecific and phylogenetic density‐dependent survival differs across life stages in a tropical forest. J. Ecol. 103:957–966.

