# Supplementary material for: Tree seedling richness, but not neighborhood composition, influences insect herbivory in a temperate deciduous forest community
Source: Ecol Evol. 2016 Aug 12;6(17):6310–9. doi: 10.1002/ece3.2336 (PMC5016651; doi:10.1002/ece3.2336)
Supplement: Supplementary file 1 — Table S1. Rank abundance tables for adult trees, seedlings ≥7 cm in height, and the 290 seedlings sampled for herbivore damage. [file ECE3-6-6310-s001.docx]

**Appendix S1:** additional species-level information for trees and seedlings.

**Table S1:** Rank abundance tables for adult trees, seedlings ≥ 7 cm in height, and the 290 seedlings sampled for herbivore damage.

| **Adult trees** | | **All Seedlings** | | **Photographed Seedlings** | |  |
| --- | --- | --- | --- | --- | --- | --- |
| **Species** | **Count** | **Species** | **Count** | **Species** | **Count** | |
| *Acer rubrum* | 91 | *Acer rubrum* | 1254 | *Fraxinus americana* | 37 | |
| *Acer saccharum* | 65 | *Fraxinus americana* | 1038 | *Nyssa sylvatica* | 29 | |
| *Prunus serotina* | 28 | *Lindera benzoin* | 537 | *Prunus serotina* | 23 | |
| *Liriodendron tulipifera* | 23 | *Prunus serotina* | 527 | *Ostrya virginiana* | 19 | |
| *Betula lenta* | 21 | *Hamamelis virginiana* | 309 | *Quercus rubra* | 19 | |
| *Betula alleghaniensis* | 17 | *Carya* spp. | 175 | *Acer pensylvanicum* | 18 | |
| *Nyssa sylvatica* | 17 | *Vaccinium angustifolium* | 145 | *Acer rubrum* | 17 | |
| *Fagus grandifolia* | 16 | *Nyssa sylvatica* | 130 | *Carpinus caroliniana* | 17 | |
| *Quercus rubrum* | 12 | *Sassafras albidum* | 128 | *Crataegus* spp. | 17 | |
| *Magnolia accuminata* | 8 | *Quercus rubra* | 121 | *Carya* spp. | 15 | |
| *Fraxinus americana* | 7 | *Fagus grandifolium* | 111 | *Fagus grandifolium* | 15 | |
| *Sassafras albidum* | 6 | *Amelanchier* spp. | 91 | *Betula lenta* | 14 | |
| *Carya ovata* | 5 | *Crataegus* spp. | 98 | *Ssassafras albidum* | 14 | |
| *Carya glabra* | 4 | *Viburnum prunifolium* | 88 | *Acer saccharum* | 7 | |
| *Quercus montana* | 4 | *Liriodendron tulipifera* | 82 | *Amelanchier* spp. | 7 | |
| *Platanus occidentalis* | 3 | *Quercus montana* | 80 | *Liriodendron tulipifera* | 7 | |
| *Robinia pseudoacacia* | 3 | *Acer saccharum* | 71 | *Cornus florida* | 4 | |
| *Tilia americana* | 3 | *Ostrya virginiana* | 70 | *Magnolia accuminata* | 4 | |
| *Acer pensylvanicum* | 2 | *Carpinus caroliniana* | 68 | *Tilia americana* | 4 | |
| *Carya cordiformis* | 2 | *Betula lenta* | 66 | *Quercus alba* | 3 | |
| *Amelanchier arborea* | 1 | *Acer pensylvanicum* | 60 |  |  | |
| *Cornus florida* | 1 | *Ulmus* spp. | 34 |  |  | |
| *Juglans nigra* | 1 | *Gaultheria procumbens* | 28 |  |  | |
| *Ulmus rubra* | 1 | *Magnolia accuminata* | 27 |  |  | |
|  |  | *Quercus alba* | 21 |  |  | |
|  |  | *Viburnum acerifolium* | 16 |  |  | |
|  |  | *Qercus velutina* | 11 |  |  | |
|  |  | *Tilia americana* | 11 |  |  | |
|  |  | *Cornus florida* | 7 |  |  | |
|  |  | *Quercus coccinea* | 5 |  |  | |

**Figure S1:** Boxplots showing the proportion of leaf tissue area removed or damaged from 290 sampled tree seedlings. The number of seedlings sampled is shown in parentheses, and species are listed in order from largest to smallest median damage.
