# Supplementary material for: Tree seedling richness, but not neighborhood composition, influences insect herbivory in a temperate deciduous forest community
Source: Ecol Evol. 2016 Aug 12;6(17):6310–9. doi: 10.1002/ece3.2336 (PMC5016651; doi:10.1002/ece3.2336)
Supplement: Supplementary file 2 — Table S2. Linear mixed‐effects models relating adult neighbor basal area, composition and rarefied species richness with proportion of leaf area lost due to herbivore damage. [file ECE3-6-6310-s002.docx]

**Appendix S2:** Model output from adult tree neighborhoods using all nine circular subplots

| **Model Formula** | **Estimate** | **S.E.** | **P-value** | **AIC** | **BIC** |
| --- | --- | --- | --- | --- | --- |
| 1) ln(Damage) ~ ln(Height) + Basal Area |  |  |  | 976.5 | 998.4 |
| ln(Height) | 0.535 | 0.290 | 0.067 |  |  |
| Basal Area | 0.060 | 0.093 | 0.523 |  |  |
| 2) ln(Damage) ~ ln(Height) + Rarefied Richness |  |  |  | 971.6 | 993.5 |
| ln(Height) | 0.508 | 0.286 | 0.078 |  |  |
| Rarefied Richness | -0.803 | 0.797 | 0.316 |  |  |
| 3) ln(Damage) ~ ln(Height) + Conspecifics + Heterospecifics |  |  |  | 978.6 | 1004.2 |
| Height | 0.540 | 0.291 | 0.066 |  |  |
| Conspecifics | 0.175 | 0.351 | 0.619 |  |  |
| Heterospecifics | 0.054 | 0.095 | 0.566 |  |  |
| 4) ln(Damage) ~ ln(Height) + Congenerics + Heterogenerics |  |  |  | 979.2 | 1004.8 |
| ln(Height) | 0.536 | 0.290 | 0.067 |  |  |
| Congenerics | -0.080 | 0.186 | 0.669 |  |  |
| Heterogenerics | 0.084 | 0.097 | 0.385 |  |  |
| 5) ln(Damage) ~ ln(Height) + Confamilials + Heterofamilials |  |  |  | 978.1 | 1003.7 |
| ln(Height) | 0.546 | 0.289 | 0.061 |  |  |
| Confamilials | -0.141 | 0.168 | 0.402 |  |  |
| Heterofamilials | 0.103 | 0.097 | 0.290 |  |  |

**Table S2:** Linear mixed-effects models relating adult neighbor basal area, composition and rarefied species richness with proportion of leaf area lost due to herbivore damage. Neighborhoods were calculated using the basal area and species richness of adult trees ≥ 8 cm DBH from nine circular 10 m radius subplots surrounding the seedling belt transects.
